# Factor associated with self-reported work-related musculoskeletal disorders in Brazilian adults

**DOI:** 10.1590/S1518-8787.2017051000282

**Published:** 2017-06-01

**Authors:** Ada Ávila Assunção, Mery Natali Silva Abreu

**Affiliations:** I Departamento de Medicina Preventiva e Social. Universidade Federal de Minas Gerais. Belo Horizonte, MG, Brasil; II Departamento de Enfermagem Aplicada. Universidade Federal de Minas Gerais. Belo Horizonte, MG, Brasil

**Keywords:** Cumulative Trauma Disorders, epidemiology, Risk Factors, Socioeconomic Factors, Working Conditions, Health Surveys, Transtornos Traumáticos Cumulativos, epidemiologia, Fatores de Risco, Fatores Socioeconômicos, Condições de Trabalho, Inquéritos Epidemiológicos

## Abstract

**OBJECTIVE:**

To describe the prevalence of work-related musculoskeletal disorder (WMSD) and analyze the factors associated with this outcome in the Brazilian population.

**METHODS:**

In this cross-sectional, population-based study, we use data from the National Survey on Health (PNS) of 2013. The sample was composed of 60,202 Brazilians aged 18 years or older. The outcome variable was the occurrence of self-reported WMSD. Sociodemographic and occupational characteristics, personal resources, and health conditions were investigated as explanatory variables. Analyses were performed with the software Stata 12.0 and considered the weighting imposed by the sampling design of the study. Then, univariate and multivariate binary logistic models were carried out, considering a significance level of 5%.

**RESULTS:**

The results obtained indicated that the prevalence of WMSD in the Brazilian population was of 2.5%, ranging from 0.2% (Acre) to 4.2% (Santa Catarina). The factors associated with a greater chance of occurrence of WMSD were: female sex (OR = 2.33; 95%CI 1.72–3.15); be temporarily away from work (OR = 2.44; 95%CI 1.41–4.23); be exposed to noise at the workplace (OR = 2.16; 95%CI 1.68–2.77); seniority equal to or greater than 4.5 years at the current job (OR = 1.37; 95%CI 1.09–1.72); participate in volunteer work (OR = 1.65; 95%CI 1.25–2.17); report medical diagnosis of arthritis or rheumatism (OR = 2.40; 95%CI 1.68–3.44); and depression (OR = 2.48; 95%CI 1.86–3.31). On the other hand, factors associated with less chance of WMSD were: not having a partner (OR = 0.73; 95%CI 0.37–0.71) and working in an open environment (OR = 0.51; 95%CI 0.37–0.71).

**CONCLUSIONS:**

The associated factors and the prevalence found indicate regional and gender differences. Special attention to comorbidities and environmental noise monitoring would benefit the health of workers in the Country.

## RESUMO

**OBJETIVO:**

Descrever a prevalência de distúrbio osteomuscular relacionado ao trabalho (Dort) e analisar os fatores associados com esse desfecho na população brasileira.

**MÉTODOS:**

Neste estudo transversal, de base populacional, utilizamos dados da Pesquisa Nacional de Saúde (PNS) de 2013. A amostra foi composta por 60.202 brasileiros com 18 anos ou mais de idade. A variável desfecho foi a ocorrência de Dort autorreferida. Como variáveis explicativas, foram investigadas características sociodemográficas, ocupacionais, recursos pessoais e condições de saúde. As análises foram realizadas com o *software* Stata 12.0 e levaram em consideração as ponderações impostas pelo delineamento amostral do estudo. Foi realizado, então, um modelo logístico binário uni e multivariado, considerando o nível de significância de 5%.

**RESULTADOS:**

Os resultados obtidos indicaram que a prevalência de Dort na população brasileira foi de 2,5%, variando de 0,2% (Acre) a 4,2% (Santa Catarina). Os fatores associados à maior chance de ocorrência de Dort foram: sexo feminino (OR = 2,33; IC95% 1,72–3,15); estar afastado temporariamente do trabalho (OR = 2,44; IC95% 1,41–4,23); estar exposto a ruído no local de trabalho (OR = 2,16; IC95% 1,68–2,77); antiguidade igual ou superior a 4,5 anos no trabalho atual (OR = 1,37; IC95% 1,09–1,72); participar de trabalho voluntário (OR = 1,65; IC95% 1,25–2,17); relatar diagnóstico médico de artrite ou reumatismo (OR = 2,40; IC95% 1,68–3,44) e de depressão (OR = 2,48; IC95% 1,86–3,31). Por outro lado, foram associados à menor chance de diagnóstico de Dort: não ter parceiro (OR = 0,73; IC95% 0,37–0,71) e trabalhar em ambiente aberto (OR = 0,51; IC95% 0,37–0,71).

**CONCLUSÕES:**

Os fatores associados e a prevalência encontrada indicam diferenças regionais e de gênero. Especial atenção às comorbidades e monitoramento do ruído ambiental beneficiariam a saúde dos trabalhadores no país.

## INTRODUCTION

Work-related musculoskeletal disorders (WMSD) refer to a range of conditions resulting from inflammation or degeneration of tendons, nerves, ligaments, muscles, and periarticular structures in different sites (fingers, wrists, forearms and arms, shoulders, and cervical region) of the upper limbs and neck^1,2^.

The terms used in the literature differ according to the social security legislation in force in each country: repetitive strain injury (RSI), cumulative trauma injuries, work-related nonspecific disorders of the upper limbs, work-related musculoskeletal problems, cervicobrachial syndrome of occupational origin, among others. What those terms have in common is that they designate localized inflammation, nerve compression syndromes, or pain syndromes. For this reason, they are considered umbrella terms^2^. The different existing nominations have already been the subject of publications in the specialized literature^3^.

In 1987, for the first time, Brazil Social Security system recognized this group of disorders with the denomination “typist’s tenosynovitis.” In 1991, the acronym RSI was adopted in internal procedures of the institution for the assessment of incapacity. In 1998, by the Technical Standard of the National Institute of Health and Social Security (INSS), approved by Service Order INSS/DSS No. 606, of August 5, 1998, formalized the use of WMSD to designate such disorders.

The acronym RSI, in Brazil, has reached the common sense since the social negotiations in the 1980s, by the recognition of the occupational nature of the disorders. Despite having inaccuracies, WMSD is more comprehensive, since symptoms may arise associated with a stress or trauma, and not exclusively due to a repetitive gesture, as suggested by the acronym (RSI) that has become more widespread. It is worth mentioning that environmental factors and tasks that require repetition and stereotyped attitudes are recognized risks.

The progress of the research and the scientific evidence produced in the last three decades is undeniable, as several authors dedicated themselves to identify the factors related to the emergence and evolution of workers’ complaints^1,2^. The results are enlightening: they do not derive from sudden injuries, neither from systemic ones, but from low intensity traumas, acting for long periods on normal or altered musculoskeletal structures. Heavy loads can change the tissues when they surpass the physical capacity of response, producing muscle or ligament injury. In its turn, lighter loads, when continuously applied, can cause inflammation, due to the prolonged stretch of the tissues from the structures required by the movement^3^. Its origin is multifactorial and evidence indicating the contribution of the organization of work in the development of these disorders is consistent^1,2^.

In the European Union, musculoskeletal disorders represent 53% of the total number of occupational diseases recorded, and 50% of the situations that lead to absences from work for over three days^4^. In the United States, these disorders are responsible for 29% of all diseases and accidents that lead to work leave^5^.

In Brazil, there are no nationwide data available with fineness of detail. However, prevalence studies allow a rough estimate of the severity of the situation. According to Social Security records, in the last decade, the diagnostic group with higher prevalence of benefits of the sickness leave-type comprised musculoskeletal diseases^a^.

Much evidence can be found in this regard. For instance, 50% of bank workers in the city of Pelotas, RS, mentioned having experienced musculoskeletal pain in the past year^6^. Among the steelworkers of the city of Canoas, RS, 75.2% reported some type of musculoskeletal symptom in the past year^7^. Using the same reference period and questioning about all body segments, the prevalence of musculoskeletal pain in a sample of health workers from the city of Salvador, BA, was 49.9%^8^. However, there are no known results of population studies. This study proposes to describe the prevalence of WMSD and to analyze the factors associated with this outcome in the Brazilian population.

## METHODS

### Study Design and Data Collection

This is a cross-sectional study, based on secondary data collected by the National Survey on Health (PNS)^b^.

The PNS is a household population-based and national level survey, carried out in partnership with the Brazilian Institute of Geography and Statistics (IBGE), from August 2013 to February 2014. It will be part of the Integrated System of Household Surveys (SIPD) of IBGE and shall have a periodicity of 5 years. This survey aims to produce data on the health status and lifestyle of the population of Brazil, as well as to assess access to and use of health services, preventive actions, continuity of care, and financing of health care.

The target population of the PNS is residents of private permanent households, and has all national territory as its demographic scope. PNS used conglomerate sampling in three stages, with stratification of the primary sampling units. The census tracts or set of tracts constitute the primary sampling units; the households were the second-stage units; and residents aged 18 years or older, third-stage units.

PNS 2013 sampling is composed by 64,348 households, in which 60,202 Brazilians aged 18 years or more accepted to answer to the individual questionnaire and, therefore, make up the sample investigated in this study.

### Studied Variables

The outcome of this study was the occurrence of self-reported WMSD, characterized by positive response to the following question: “Has some doctor ever diagnosed you with WMSD (work-related musculoskeletal disorder)?” Age at first diagnosis, treatment used, and the level of limitation of activity due to the disease were also considered in the assessment.

Regarding the factors associated with the occurrence of WMSD, three blocks of variables were analyzed: 1. sociodemographic characteristics (sex, age, marital status, and education level); 2. occupational characteristics (work paid in money, work paid in products, “odd jobs” or occasional activities, unpaid work, temporary leave from work, time and reason for the leave, numbers of jobs or equivalent) and working conditions (night hours, open or closed environment, exposure to noise, seniority in the current employment); and 3. personal resources and health conditions (participation in sport activities, the neighborhood association, volunteer work, or cults and religious activities, engaging in physical activity, health assessment, report of having been diagnosed with arthritis and depression, use of medication to sleep).

### Statistical Analysis

All analyses were performed with the software Stata 12.0 and considered the weighting imposed by the sampling design of the study.

Initially, a descriptive analysis of all the studied variables was made by calculating relative frequencies and constructing a bar graph. To assess the possible factors associated with the occurrence of WMSD, Pearson’s Chi-square test was used, as well as the binary logistic regression model, both univariate and multivariate.

For the variables to be entered in the multivariate analysis, a p-value of less than 0.20 was considered in the univariate analysis. Models were constructed for each block of variables (sociodemographic characteristics; occupational characteristics, including working conditions; personal resources; health conditions) using the backward method to enter the variables in each block. In the final model of each block remained variables with p < 0.05. Then, the final model was adjusted by the variables of all blocks, remaining only those with significant level of 5%. The odds ratio (OR) value were estimated, with 95% confidence interval (95%CI), both in the univariate and multivariate analyses. The goodness of fit was assessed by Hosmer and Lemeshow statistics.

PNS was approved by the National Research with Human Beings Ethics Committee (Conep – Process: 328.159, on June 26, 2013).

## RESULTS

The prevalence of WMSD in the Brazilian population was of 2.5% (95%CI 2.2–2.7), ranging from 0.2% (Acre) to 4.2% (Santa Catarina) (Figure). Among the respondents who reported the diagnosis, 25.5% did physical exercise or physical therapy due to the disease, 35.7% used WMSD drugs, and 2.6% did acupuncture. More than half of the respondents reported that the disorders limited their usual activities – for 13.1% of them this limitation was intense and to 2.8%, very intense. The average age when diagnosed with WMSD was 36.3 years (95%CI 34.9–37.8).

**Figure:**
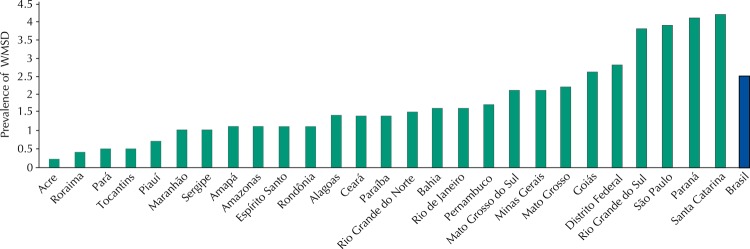
Prevalence of WMSD in the Brazilian population in each of the 26 states and the Federal District, according to data from the PNS, Brazil, 2013.

WMSD: work-related musculoskeletal disorders.

Regarding the characteristics of the sample, women prevailed (55.1% of respondents); 27.1% were aged 55 years or more; 54.5% did not have a partner; and 35.5% reported having elementary education level. In relation to occupational characteristics and working conditions, 56.9% of the respondents were paid in money; 0.2% with products or services; 1.8% had “odd jobs” or worked occasionally; and 0.8%, despite working, were not paid. About work leaves, 1.5% were temporarily away (of these, 31.0% were provided with benefits); 46.2% were related to an occupational disease; and 56.9% for a period of three months or more. The category “having more than one job” was reported by 2.6% of Brazilians and, of these, 14.9% worked at night; 52.3% in closed environments; and 32.1% were exposed to noise in the workplace. As for seniority, the median was 4.5 years.

As for personal resources and health conditions, 25.4% of respondents reported participating in sport or artistic activities, 16% participated of neighborhood association, 12.1% practiced volunteer work, and 70.4% attended cults and religious celebrations. The prevalence of the practice of physical activity was of 31.0%. The majority (52.5%) of the Brazilians assessed their health as good. The prevalence of arthritis or rheumatism was 6.7%, while depression was 7.9%. Of the total of respondents, 8.0% reported using medication to sleep.

According to the univariate analysis, sociodemographic factors (Table 1) associated with higher prevalence of WMSD were: female sex, age ranging from 25–54 years, living without a partner, and higher education level (p < 0.05). Regarding occupational factors (Table 2), those that were significantly associated with greater chance of occurrence of WMSD were: to inform work paid in money, be temporarily away (due to occupational disease), have two or more jobs, work in closed environments, be exposed to noise at work, and seniority equal to or greater than 4.5 years. As for personal resources and health conditions (Table 3) associated with a greater likelihood of WMSD (p < 0.05), the following factors were identified: participate in sport or artistic activities, participate in neighborhood association, participate in volunteer work or cults or religious activities, report regular or poor health condition, report diagnosis of arthritis or rheumatism and depression, and use medication to sleep.

**Table 1:** Univariate analysis assessing sociodemographic factors associated with the occurrence of WMSD, according to data from the PNS, Brazil, 2013.

Variable	Percentage (%) total sample	Prevalence (%) of WMSD	p*	OR	95%CI
Sex					
Male	44.9	1.5	< 0.001	1.00	
Female	55.1	3.3		2.22	1.80–2.75
Age group (years)					
18–24	14.5	1.3	< 0.001	1.00	
25–34	21.0	2.3		1.78	1.11–2.87
35–44	19.2	3.3		2.59	1.63–4.11
45–54	18.2	3.7		2.93	1.85–4.66
≥ 55	27.1	1.9		1.43	0.86–2.37
Marital status					
Have a partner	45.1	3.0	< 0.001	1.00	
No partner	54.9	2.1		0.67	0.56–0.80
Education level					
Illiterate/no formal education	13.9	1.7	< 0.001	1.00	
Elementary school	35.5	2.3		1.41	1.01–1.96
High school	33.0	2.5		1.53	1.09–2.14
Higher education	17.5	3.5		2.18	1.48–3.21

WMSD: work-related musculoskeletal disorders.

* Pearson’s Chi-square test.

**Table 2:** Univariate analysis assessing occupational characteristics and work conditions associated with the occurrence of WMSD, according to data from the PNS, Brazil, 2013.

Variable	Percentage (%) total sample	Prevalence (%) of WMSD	p^a^	OR	95%CI
Work paid in money					
No	43.2	2.1	0.005	1.00	
Yes	56.9	2.8		1.34	1.09–1.64
Work paid in products					
No	99.8	2.5	0.733	1.00	
Yes	0.2	3.5		1.42	0.19–10.56
“Odd jobs” or occasional activities					
No	98.2	2.5	0.942	1.00	
Yes	1.8	2.4		0.97	0.48–1.98
Unpaid work					
No	99.2	2.5	0.581	1.00	
Yes	0.8	1.9		0.75	0.27–2.10
Temporary leave from work					
No	98.5	2.4	< 0.001	1.00	
Yes	1.5	9.6		4.34	2.92–6.45
Paid work leave					
No	69.0	7.6	0.305	1.00	
Yes	31.0	2.7		0.33	0.03–3.51
Occupational disease-related work leave					
No	53.8	4.5	0.002	1.00	
Yes	46.2	28.3		8.47	1.93–37.13
Time of work leave					
Up to 15 days	9.6	10.1	0.347	1.00	
More than 15 days and less than 3 months	33.5	18.5		2.01	0.37–10.86
3 months and more	56.9	10.2		1.01	0.18–5.65
How many jobs					
None	39.5	1.9	< 0.001	1.00	
One	57.9	2.8		1.47	1.18–1.83
Two or more	2.6	5.0		2.73	1.69–4.39
Work at night hours					
No	85.3	2.8	0.568	1.00	
Yes	14.7	3.1		1.10	0.79–1.55
Works on environments					
Closed	52.3	3.7	< 0.001	1.00	
Open	27.5	1.5		0.38	0.28–0.53
Both	20.2	2.7		0.71	0.52–0.95
Exposure to noise at the workplace					
No	67.9	2.3	< 0.001	1.00	
Yes	32.1	4.1		1.79	1.43–2.24
Seniority at the current job^b^					
Up to 4.5 years	49.9	2.4	0.005	1.00	
More than 4.5 years	50.1	3.3		1.37	1.10–1.72

WMSD: work-related musculoskeletal disorders.

^a^ Pearson’s Chi-square test.

^b^ Median = 4.5 years.

**Table 3:** Univariate analysis assessing personal resources and health conditions associated with the occurrence of WMSD, according to data from the PNS, Brazil, 2013.

Variable	Percentage (%) total sample	Prevalence (%) of WMSD	p*	OR	95%CI
Participated in sport or artistic activities					
No	74.6	2.3	0.020	1.00	
Yes	25.4	3.0		1.27	1.04–1.56
Participated in neighborhood association					
No	84.0	2.4	0.028	1.00	
Yes	16.0	3.0		1.27	1.03–1.57
Participated in volunteer work					
No	87.9	2.2	< 0.001	1.00	
Yes	12.1	4,7		2.20	1.74–2.78
Participated in cults and religious activities					
No	29.6	1.7	< 0.001	1.00	
Yes	70.4	2.9		1.73	1.38–2.18
Practiced some type of physical activity					
No	69.0	2.4	0.315	1.00	
Yes	31.0	2.7		1.11	0.90–1.38
Reported health					
Very good	13.0	2.4	0.001	1.00	
Good	52.5	2.1		0.87	0.63–1.19
Regular, poor and very poor	34.6	3.1		1.29	0.94–1.77
Diagnosis of arthritis or rheumatism					
No	93.3	2.2	< 0.001	1.00	
Yes	6.7	6.3		2.96	2.31–3.81
Diagnosis of depression					
No	92.1	2.1	< 0.001	1.00	
Yes	7.9	7.3		3.67	2.96–4.55
Uses medication to sleep					
No	92.0	2.3	< 0.001	1.00	
Yes	8.0	4.9		2.22	1.72–2.87

WMSD: work-related musculoskeletal disorders.

* Pearson’s Chi-square test.

According to the final model of the multivariate analysis (Table 4), the factors associated with a greater chance of occurrence of WMSD were: female sex; be exposed to noise at the workplace; seniority equal to or greater than 4.5 years at the current job; participating in volunteer work; report medical diagnosis of arthritis or rheumatism and depression. Two factors associated with less chance of WMSD stood out: not having a partner and working in an open environment. It should be noted that the model presented good fit according to Hosmer and Lemeshow statistics (p = 0.962).

**Table 4:** Multivariate analysis (binary logistic regression model) assessing factors associated with the occurrence of WMSD, according to data from the PNS, Brazil, 2013.

Variable	p*	OR	95%CI
Sex			
Male		1.00	
Female	< 0.001	2.33	1.72–3.15
Marital status			
Have a partner		1.00	
No partner	0.006	0.73	0.58–0.91
Temporary leave from work			
No		1.00	
Yes	0.001	2.44	1.41–4.23
Works on environments			
Closed		1.00	
Open	< 0.001	0.51	0.37–0.71
Both	0.139	0.78	0.56–1.08
Exposure to noise at the workplace			
No		1.00	
Yes	< 0.001	2.16	1.68–2.77
Seniority at the current job			
Up to 4.5 years		1.00	
More than 4.5 years	0.007	1.37	1.09–1.72
Participated in volunteer work			
No		1.00	
Yes	< 0.001	1.65	1.25–2.17
Diagnosis of arthritis or rheumatism			
No		1.00	
Yes	< 0.001	2.40	1.68–3.44
Diagnosis of depression			
No		1.00	
Yes	< 0.001	2.48	1.86–3.31

WMSD: work-related musculoskeletal disorders.

* Hosmer and Lemeshow goodness of fit statistics = 0.962.

## DISCUSSION

PNS is the first nationwide study to investigate the self-reported prevalence of an occupational disease. WMSD stands out in the ranking of reasons that lead adult workers in Brazil, as well as in other industrialized countries, to miss work days and be disabled.

Sex, seniority in current work equal to or greater than 4.5 years, temporary leave from work, report of exposure to noise at the workplace, participation in voluntary unpaid work, report having been diagnosed with arthritis by a physician, and report having been diagnosed with depression by a mental health professional have been associated with a greater chance of occurrence of WMSD. Living without a partner and working in open environment decreased the chances of occurrence of the disorders.

The disease was reported by 2.5% of respondents in Brazil, varying from 0.2% to 4.2%. It is not possible to establish comparisons ideals, because, although the results about the magnitude and the factors associated with musculoskeletal morbidities in specific populations are described, population-based studies that focused on musculoskeletal illness specifically related to work are rare.

Verifying the prevalence differences when comparing the Brazilian states provides an opportunity for discussing the social inequalities and the geographical distribution of health services in the Country, which affect access to care. It is possible that the residents of the North and Northeast regions, where lower prevalence of WMSD was found, face access barriers when compared to the states of the other major regions, which would reflect on the self-reported diagnosis of WMSD and other morbidities.

In Brazil, similarly to other countries, the distribution of morbidity is characterized by social and geographic gradients. We observed that access to health services depends on where the individual lives, with advantage to regions with greater socioeconomic development^9^. The deployment of the Integral Care to Worker’s Health Network (Renast) is the main strategy of SUS national policy on worker’s health. Among the difficulties found for the consolidation of Renast, it is possible to mention its regionalized performance logic, different from SUS organization, which is city-based^10^, with possible reflections on information and registration of morbid events.

Other direction is possible to interpret the regional differences in the self-report of WMSD. The unequal offer of SUS services in the national territory is proved, with losses to lower-income groups, which coincide with the portions of the population inhabiting municipalities struggling to fulfill their responsibilities in the financing and provision of health services. The restrictions of the most poor individuals in paying for additional assistance reinforces the hypothesis about the effects of income distribution on access to care, increasing the likelihood of bias of information as to the diagnosis of morbidities^11^.

Despite the possibility of underestimation of self-reported information, WMSD is a framework originally described according to its relationship with work. It is plausible to assume that the answer to the question that generated the outcome variable leaves little margin for subtext, thus decreasing the likelihood of information bias. On the other hand, if the information bias is less likely, the same cannot be said about the controversies^3^ in terms of the occupational-clinical evaluation that would have accentuated the tendency toward underestimation of the studied prevalence. The controversy originates in the multifactorial nature of the disorders, particularly concerning the weight of individual factors. The dissent is enhanced by the existence of clinical profiles of distinct nature, patients who do not show objective signs when examined, and failures when the approach is made by only one physician, instead of by a multidisciplinary team.

Generally, the specific cases, whose pain is located and confirmed by examination using the known provocative tests (carpal tunnel syndrome, tenosynovitis, and impingement syndrome), are diagnosed in the first consultation. The cases raise controversy when the pain is apparently not explained by detectable injury or is associated with an injury that, in theory, would be insufficient to explain the intensity of the symptom. The greater chance of WMSD related to the information of being in a temporary work leave increases the consistence of the presented results. It is known that these disorders are incapacitating^7,12^.

The social iatrogenesis caused by the judiciary system when benefiting patients in cases of labor lawsuits, by Union militancy that encourages such measures, and by health systems that are “susceptible” to the diagnosis is being discussed^13^. Acting in this way, society would be feeding the polymorph clinical picture of patients who have a profile of making multiple complaints. The possibility of social iatrogenesis (fairly widespread in Australia) achieved a broad repercussion in Brazil. Verthein and Gomez (2001)^14^ exposed a substantiated criticism to what they called “the discursive bases of the neuropsychiatrization of RSI.” According to the authors, the iatrogenic ideas of disease and the simulation of patients serve to set up a network of alliances formed to deny the nexus. In this context, once again, it is plausible to assume that the prevalence found in the Brazilian population is underestimated.

In general, the effects of gender differentials^15^ when examining the prevalence of musculoskeletal disorders in the general population and in specific occupational groups are well documented. Social roles assigned to men and women affect time management: women occupy themselves with household tasks at the expense of their personal interests, while men develop sports and leisure activities in their free time^16^. In addition, the working conditions of men and women are not equal, i.e., the effects of working life are more pronounced for women because they are more vulnerable to precarious employment, receive lower wages, have lower level positions in the hierarchy, as well as lower social recognition^15^. Arguments in the biological and behavioral plan, as differences in height, muscle strength, aerobic capacity, hormonal conditions, among others, would make a woman more susceptible to musculoskeletal disorders. However, the excessive prevalence in women of musculoskeletal pain in upper limbs was explained by increased exposure to risk factors in work and household environments, to the detriment of women’s biological vulnerability to the mentioned risks^17^.

The arguments mentioned above can be evoked to explain the smaller chance of occurrence in the group living without a partner, since household tasks would be more likely in life as a couple, sometimes with children, and the resulting care for this situation. Family bonds offer support by sharing emotions, but can generate an accumulation, given the vicissitudes faced by spouses^17^. It is valid to remember that the effects of the work overload may add to the weight of family responsibilities, thus restricting the necessary pauses for rest and recovery of musculoskeletal wear^16^.

The group of individuals with greater seniority in the job had more chance to report WMSD. Seniority is a marker of duration of exposure to occupational factors, which also favors the gaining of experience. On the other hand, a decrease in the capacity of adjusting the workload is expected, due to the accumulation of effects of environmental and psychosocial factors that individuals face along their professional career^18^. Therefore, the greater chance of WMSD in this group would not be unexpected.

As for the lesser chance of occurrence in the group who reported working in open environments, other information would be necessary to develop grounded hypotheses. Still, it is not unlikely that working in this condition is free of the injunctions that characterize the industrial production management methods, which expose individuals to the repetition of cadenced operations and poor autonomy, both well documented characteristics regarding their relationship with WMSD^2,12^.

Exposure to intense noise during labor activity increased the chance of occurrence of WMSD. This result is not surprising, since the association between musculoskeletal disorders and environmental factors are known^19^. Regarding noise, we can suggest the hypothesis of extra-auditory effects, such as irritation and discomfort^20^, which cause reactions at the muscle fibers. In situations of involuntary increase of myofibrils contraction, the microbreaks needed for muscle work decrease, with losses on the reperfusion of the peritendinous tissues. Inflammatory processes resulting from this situation would explain the symptoms of muscle fatigue, pain, and, if the environmental exposure is maintained, function limitation^19^.

The greatest chance of WMSD in the group who reported participating in volunteer work in the community is consistent with the inability that the pain situation provokes. If so, individuals who had limitations to keep their jobs would be available to act in other spheres of life, for example, community actions of voluntary character. Interdisciplinary research shows important pathways to understand the impact of an incapacitating occupational disease over the different dimensions of social life^14^.

As for the report of having been diagnosed with arthritis by a physician, the prevalence found (6.7%) was lower than the results (18.53%) collected by interviews in the households of southern Asia^21^. It is possible that the difference is explained by the methodological strategy used in the PNS, whose question led to variable “evokes medical diagnosis.” However, in both cases, the prevalence of the disease is high.

A population-based survey sought to identify the duration of comorbidities in the group that reported musculoskeletal disorders in the cervical and lower back regions^22^. The prevalence of arthritis along with isolated cervical pain and cervical pain simultaneously with low back pain was of 35.1% and 46.2%, respectively, as opposed to the prevalence of 13.8% in the group that denied cervical and low back muscle pains. In agreement with the results from PNS, in the case of depressive symptoms, the same trend was verified: 23.9% and 40.1% of simultaneous self-reported prevalence, respectively, to isolated cervical pain and cervical pain along with low back pain^22^.

The maintenance of employment and work activity is a stimulated measure in rheumatological practice. Despite the benefits, the workplace can be a source of occupational stress^23^, having negative effects on the situation of individuals suffering from these rheumatic morbidities. We observed that the expected protective effect of labor activity on the evolution of the symptoms of arthritis was frustrated when the individuals were exposed to tasks for which they had restricted control margins (opening to choose when and how to perform the tasks), as opposed to high demands (volume of tasks, speed with which they should be finished, and time available to meet these requirements). These work-related tensions can be expressed as greater or lesser job satisfaction, and these experiences can lead to stressful events, which interact with the physical and mental health of the individual, thus worsening rheumatic and depression symptoms. In this sense, the PNS results that indicate these comorbidities are plausible.

Depression is an independent risk factor for a set of musculoskeletal disorders^24^. According to the hypothesis of cognitive vulnerability, stressful events tend to precipitate depressive situations^25^. The multifactorial character, as well as the role of psychosocial factors (such as dissatisfaction) in the development of musculoskeletal disorders and psychiatric comorbidities are very clear, both in the triggering and worsening of chronic musculoskeletal pain, which is the main symptom of WMSD^26^.

However, the proposed hypotheses do not allow more incursions. The report of diagnosis of depression can be associated with the investigated outcome. It is also possible, however, that the experiences related to an occupational disease are exacerbating pre-existing problems, or that they are more easily noticed by people with depressive profiles and, therefore, are more often reported by them.

The limits derived from the lack of methodological consensus indicate caution in interpreting the results. The definition of the event to characterize the outcome varies in current literature, with losses to attempts at comparison of the obtained results. It is possible to question about complaints^5-8,12,17,22^; or diagnosis^21^; to investigate musculoskeletal morbidities in general the group of workers active in a particular branch of production^6-8,12^; or interview employed individuals in their households^21^, as is also the case of PNS.

The macroeconomic structure, the internationalization of investment, and the application of new technologies can transform the policies of employment and working conditions. Considering the weakening of Union power and unemployment, the productive restructuring environment becomes conducive to the implementation of management strategies that increase stressful situations (absence of pauses, demand for more parts per unit of time, for example), as well as generate new forms of insertion of individuals on production^12,15^. In this context, the sources of information on employment, work, and health of workers become even more precarious. For the first time in Brazil, it was possible to examine the prevalence of a disease of such importance for the already discussed reasons. Memory bias, differences in estimates of illness, and inadequacy of resources to assess the quality of data collection are inherent limitations to investigations. However, the contribution of this kind of study to improve quality of care is undeniable, being thus possible to enhance programs and evaluate the policies specific to occupational groups.

In addition, it is worth noting that the statistical analysis benefited from the advantages concerning the expressive sampling size, as well as the high response rate. The sampling design of the PNS, as well as the weights used in the analysis, ensures data representativeness for Brazil, its major regions, states, metropolitan areas, capital cities and other municipalities, thus allowing an accurate characterization of the health conditions of the Brazilian population. It is known that the prevalence measures of occupational diseases are a challenge for the countries where health information systems of workers do not cover the total employed population or presents vices related to the exclusive coverage of the formally employed population, as is Brazil’s case.

The results indicate the need to monitor comorbidities in the case of musculoskeletal diseases, and strengthen programs focused on gender differentials. Special attention environmental noise monitoring would benefit the health of workers in the Country.

## Footnotes

a Ministério da Previdência Social (MPS). Anuário Estatístico da Previdência Social. Brasília: MPS; 2013.

b Instituto Brasileiro de Geografia e Estatística (IBGE). Pesquisa Nacional de Saúde 2013, IBGE. Percepção do estado de saúde, estilo de vida e doenças crônicas. Brasil, Grandes Regiões e Unidades da Federação. Rio de Janeiro: IBGE; 2014.
